# Determinants of the Perceived Credibility of Rebuttals Concerning Health Misinformation

**DOI:** 10.3390/ijerph18031345

**Published:** 2021-02-02

**Authors:** Yujia Sui, Bin Zhang

**Affiliations:** School of Economics and Management, Beijing University of Posts and Telecommunications, Beijing 100876, China; binzhang@bupt.edu.cn

**Keywords:** social media, health misinformation, rebuttal, structural equation model

## Abstract

Users provide and share information with a broad audience on different forms of social media; however, information accuracy is questionable. Currently, the health information field is severely affected by misinformation. Thus, addressing health misinformation is integral for enhancing public health. This research can help relevant practitioners (i.e., government officials, medical and health service personnel, and educators) find the most effective correctional interventions for governing health misinformation. We constructed a theoretical model for credibility-oriented determinants refuting misinformation based on the elaboration likelihood model. We aggregated 415 pieces of valid data through a questionnaire survey. A partial least squares structural equation model evaluated this research model. The results indicated that both perceived information quality and perceived source credibility can enhance perceived information credibility. Under some circumstances, the influence of information quality on information credibility may be more important than that of the information source. However, the cognitive conflict and knowledge self-confidence of information receivers weaken the influence of information quality on information credibility. In contrast, cognitive conflict can strengthen the influence of source credibility on information credibility. Further, perceived information quality can be affected by information usefulness, understandability, and relevance, while perceived source reliability can be affected by source expertise and authority.

## 1. Introduction

The existence and transmission of health misinformation have led to severe consequences to personal health, social media platform operation, and social stability [1,2]. For instance, when the Zika virus broke out, misinformation related to this virus attracted widespread attention on Facebook and was more popular than correct, reliable information [3]. The expenditure for launching unnecessary informational promotion activities to correct such misinformation grew significantly [4]. Furthermore, in the wake of the COVID-19 outbreak, more than 600 people died in Iran after they drank high levels of alcohol in the mistaken belief that it would protect them against the virus [5,6]. Hence, the public’s belief in misinformation leads to more dangerous consequences than ignorance [4].

In recent years, the Internet has become the main source of information, and with a surge in demand for health information, most people choose to browse the Internet to obtain it. According to a report published by the Pew Research Center, 72% of adults in the US have searched for at least one type of health information on the Internet [7]. Because of its convenience, the Internet meets the public’s demands for having face-to-face consultations with professional medical and nursing personnel [8]. Nonetheless, it is also responsible for the prevalence of health misinformation [9], because traditional quality-control mechanisms, such as professional editors, are excluded from the information-generation process. Particularly, the development of Web 2.0 has changed Internet users from passive information consumers to users who actively generate content on websites such as Weibo, Zhihu, and YouTube [4]. Misinformation is consequently widely transmitted and starts trending on social media platforms [10]. Notably, health information is one of the information sources that attracts the most attention but is most severely affected by misinformation [11].

UNESCO, in its working documents and reports, defines misinformation as unintentional misinformation disseminated with confidence of its authenticity, usually with no apparent intention of profit behind it [12,13]. The consensus of the scientific community provides a relatively clear distinction between true information and misinformation. Health misinformation, which is the focus of this study, is contrary to the cognitive consensus of the scientific community on a certain phenomenon [14]. There are three modes of handling misinformation. The first mode is preemptive prevention. In this mode, true information is transmitted to the public before it is subject to misinformation. Therefore, relevant studies focus on the acceptance of health information [15]. The second mode utilizes the withdrawal or deletion of misinformation. Some studies have prioritized the identification of misinformation and discontinution of its dissemination in time [16]. Although this mode is conducive to mitigating the impact of misinformation, it does not eliminate it [17]. The third, and often the most effective, mode when individuals pursue accuracy motivational goals in processing scientific information includes explanation and correction. This mode is the focus of this study. Correction provides information about beliefs that individuals may hold, stemming from previous contact and communication [18].

The public’s belief in misinformation leads to more dangerous consequences than ignorance [4]. Thus, to successfully right users’ cognitive errors, corrective health information must convince them. This study differs from past studies in terms of information adoption, because correcting misinformation means the original beliefs held by information recipients must be changed. Misinformation rebuttals are messages telling individuals to ignore or disbelieve previous information [18]. In other words, persuasive knowledge is required for information recipients under high-level cognitive conflicts. Additionally, professional knowledge is needed for such refusal of health misinformation to occur, because it is more difficult for the public to cognitively process health information than other types of information. To our knowledge, no prior study evaluated the impact of information recipients’ cognitive conflicts and knowledge self-confidence while refuting health misinformation.

This study aims to explore an effective way to correct health misinformation and eliminate its adverse effects. Although some information on social media can be misleading, or even deceptive, the more people feel they can trust health information, the more they are willing to accept it [19,20]. We constructed a theoretical model for credibility-oriented determinants refuting misinformation based on the elaboration likelihood model. The partial least squares (PLS) method was used to verify the proposed model. The findings of this study complement previous research on social media and online health. Alternatively, this study can help relevant practitioners (i.e., government officials, platform managers, medical and health service personnel, and educators) find the most effective correctional interventions for governing health misinformation.

## 2. Research Model and Hypotheses

The rebuttal of misinformation falls in the category of persuasion information, which aims to correct the public’s misunderstandings and provide knowledge about the truth of matters. The elaboration likelihood model (ELM), proposed by psychologists Petty and Cacioppoti, is one of the most authoritative theories in the field of knowledge persuasion [21]. It has been widely used to describe how people process information and form their attitudes toward behaviors. According to the ELM, persuasion can be achieved through one or both of the following routes: the central and peripheral routes. The central route of persuasion focuses on information factors. In this route, the information recipient inputs a large number of cognitive resources for the elaborate processing of information to produce the perception of contacted information [22]. In comparison, the peripheral route of persuasion focuses on irrelevant factors, such as the source and presentation of information. In this route, the recipient processes information on the low level [23]. Both routes signify that one’s attitudes take shape or vary according to intrinsic information processing capabilities [24]. Previous empirical studies have verified the application of the ELM in various fields. For example, the ELM and technology acceptance model (TAM) can be combined to study how knowledge-based employees evaluate information and accept advice [24]. Chung et al. studied the adoption of tourist information on social media through the ELM and explored the moderating roles of social presence [25]. Tseng and Wang investigated the information adoption process on tourist websites regarding cognitive risks through the integrated model of ELM and perceived usefulness [26].

According to the three main factors of the effectiveness of information transmission, the influencing factors of the credibility of refusals can be divided into the information source, the information itself, and the information receiver. Based on the ELM, this study constructs a structural equation model for relevant determiners. Specifically, information quality is used as the central route, while source credibility is regarded as the peripheral route. As for the recipient, we explore the moderating role of the public’s cognitive conflicts and knowledge self-confidence in central and peripheral routes.

### 2.1. Perceived Information Quality

Perceived information quality is defined as the values and proof persuasion of information [24,27,28]. According to the application of the ELM on information adoption, information quality affects one’s attitudes through the central route. Information quality affects the degrees of perceived usefulness [24,27] and trust [21] in information, as well as users’ attitudes and willingness [29]. High-quality information has a significant impact on the persuasion effect [30], and it plays a role in changing people’s attitudes, even when they are concerned about privacy [22]. Conversely, low-quality, irrational, and non-persuasive information has no significant impact on recipients’ attitudes [31]. Excessive advertisements and misleading health information on social media make it more difficult for users to identify whether the information is true or false. Health information with high perceived quality would have an increasingly vital role in determining people’s trust in health information [15]. Typically, the information quality of Internet health information is generally affected by relevance, understandability (i.e., clarity and readability), adequacy (i.e., sufficiency, completeness, and necessity), and usefulness [29,32]. The following hypotheses are put forward based on existing studies:

**Hypothesis** **1** **(H1):** *Perceived information quality would have a positive relationship with the perceived credibility of rebuttals concerning health misinformation.*

**Hypothesis** **1a** **(H1a):** *Information relevance would have a positive relationship with perceived information quality.*

**Hypothesis** **1b** **(H1b):** *Information understandability would have a positive relationship with perceived information quality.*

**Hypothesis** **1c** **(H1c):** *Information adequacy would have a positive relationship with perceived information quality.*

**Hypothesis** **1d** **(H1d):** *Information usefulness would have a positive relationship with perceived information quality.*

### 2.2. Perceived Source Credibility

Source credibility refers to the degree of credibility of the information sender as perceived by the information recipient [23]. Thus, it represents an attitude toward the information source and is irrelevant in terms of the information itself [33]. According to questionnaire survey and experimental study, the impact of perceived source credibility on people’s attitudes and information accepting behaviors is widely accepted. This notion affects the adoption of tourist information from user-generated content on social media [25] and the evaluation of online health information [34]. Source credibility plays a vital role in improving users’ experiences and enhancing their behavioral intentions in the virtual community [35]. Individuals are more inclined to believe information from a highly reliable source rather than a source with low reliability [36]. The misinformation rebuttals on the Internet do not exist independently but are overshadowed by a large number of true or false information flows [37]. Understandably, source credibility allows people to handle information through the peripheral route rather than rely on complicated cognitive processing [21]. Many virtual communities infer the credibility of a knowledge source through the user influence system based on historical contributions and published records [38]. The user’s authority is valid for judging the source credibility of microblog information [39]. The recipient’s decision-making process will be more affected by the provider if the knowledge provider has a high level of professional knowledge [40]. The perceived source reliability of health information mainly depends on the expertise (i.e., competence, skill, and knowledge) and authority (i.e., reputation, status, and influences) of information publishers as perceived by the public. The following hypotheses are put forward based on existing literature:

**Hypothesis** **2** **(H2):** *Perceived source credibility would have a positive relationship with the perceived credibility of health misinformation rebuttals.*

**Hypothesis** **2a** **(H2a):** *Source expertise would have a positive relationship with perceived source credibility.*

**Hypothesis** **2b** **(H2b):** *Source authority would have a positive relationship with perceived source credibility.*

### 2.3. Moderating Effect of Cognitive Conflict

Regarding the persuasion field, many studies have shown that people accept information more easily when it is consistent with what they consider to be correct. The consensus not only enhances users’ trust in provided information but also effectively influences the recipient’s opinions, attitudes, and beliefs [41]. After receiving new information, people immediately evaluate whether it is compatible with the logic of other facts and cognitive beliefs. If the information conflicts with the original perception, people are more likely to resist changing their original beliefs [42,43]. Therefore, conflicts with original perception may make it less likely to successfully correct misinformation.

If the information is inconsistent with one’s beliefs, it may trigger negative emotions. Further, examining information inconsistent with one’s beliefs is not as smooth as evaluating information consistent with those beliefs. Typically, conveniently deciphered information is more familiar and more easily accepted. Conversely, inconvenience triggers negative feelings and urges people to examine the information more carefully [44,45]. This process requires more effort, motivation, and cognitive resources [4]. Consequentially, such people may seek help from transmitters’ evaluations of reliability. When the information received by consumers counters their preconceived perceptions, stronger correlations between emotional trust and behavioral intentions are formed [46]. The following hypotheses are proposed based on existing studies:

**Hypothesis** **3a** **(H3a):** *Cognitive conflict would moderate the relationship between perceived information quality and perceived information credibility.*

**Hypothesis** **3b** **(H3b):** *Cognitive conflict would moderate the relationship between perceived source credibility and perceived information credibility.*

### 2.4. Moderating Effect of Knowledge Self-Confidence

The impact of the perceived information quality on one’s attitudes varies according to situations and is affected by personal abilities in specific circumstances [47]. Knowledge self-confidence refers to a self-assessment of the degree to which individuals think they understand relevant scientific knowledge [48,49]. Perceived information quality is a subjective evaluation of information content and depends on the individual’s previous experience and professional knowledge [21]. Both one’s knowledge and skills can be employed to handle information. In some cases, the content of information is read, processed, and considered, and in other cases, the content may be neglected entirely. Such differences may result from recipients’ different interpretations of knowledge content [24,50]. In the peripheral route, impacts are mainly created through simple decision-making standards and clues such as reputation, charisma, or appeal [22]. Individuals may use such clues because they do not want to invest necessary cognitive resources or do not make an effort owing to limited capacities. When judging the authenticity of information through source credibility, users need not have complicated cognitive processing for strongly professional health information. Non-expert users are more inclined to rely on what are known as marginal clues (i.e., source credibility) [51,52]. Evaluating the credibility of health misinformation rebuttals requires more professional knowledge. Source credibility may be the most pivotal factor for non-experts to evaluate information [24]. Hence, the following hypotheses are proposed based on existing studies:

**Hypothesis** **4a** **(H4a):** *Knowledge self-confidence moderates the relationship between perceived information quality and perceived information credibility.*

**Hypothesis** **4b** **(H4b):** *Knowledge self-confidence moderates the relationship between perceived source credibility and perceived information credibility.*

**Figure 1:**
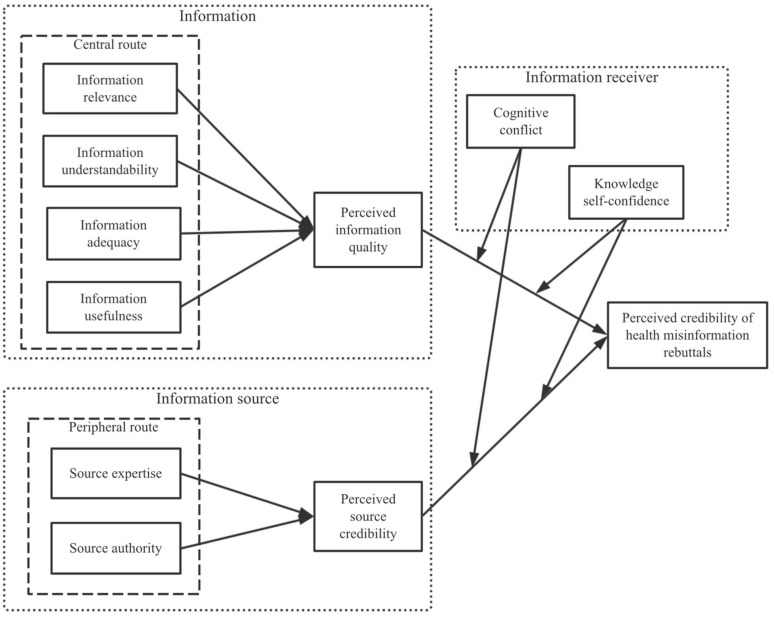
Research model.

Based on the analysis above, this study presents the research model illustrated in Figure 1.

## 3. Methods

This study cited the example of rebuttals of health misinformation on the Sina microblogging platform. The PLS structural equation model was used to verify the hypothesis model. The model structure is characterized as reflective first order.

The questionnaire comprised three parts. The first part collected respondents’ personal information and studied respondents’ original understanding of one type of health information, namely, judging whether there are cognitive mistakes. We asked participants, “Do you think bone soup can supplement calcium?” The respondents answering with “Yes” (cognitive errors) were screened into the second and third parts.

**Figure 2:**
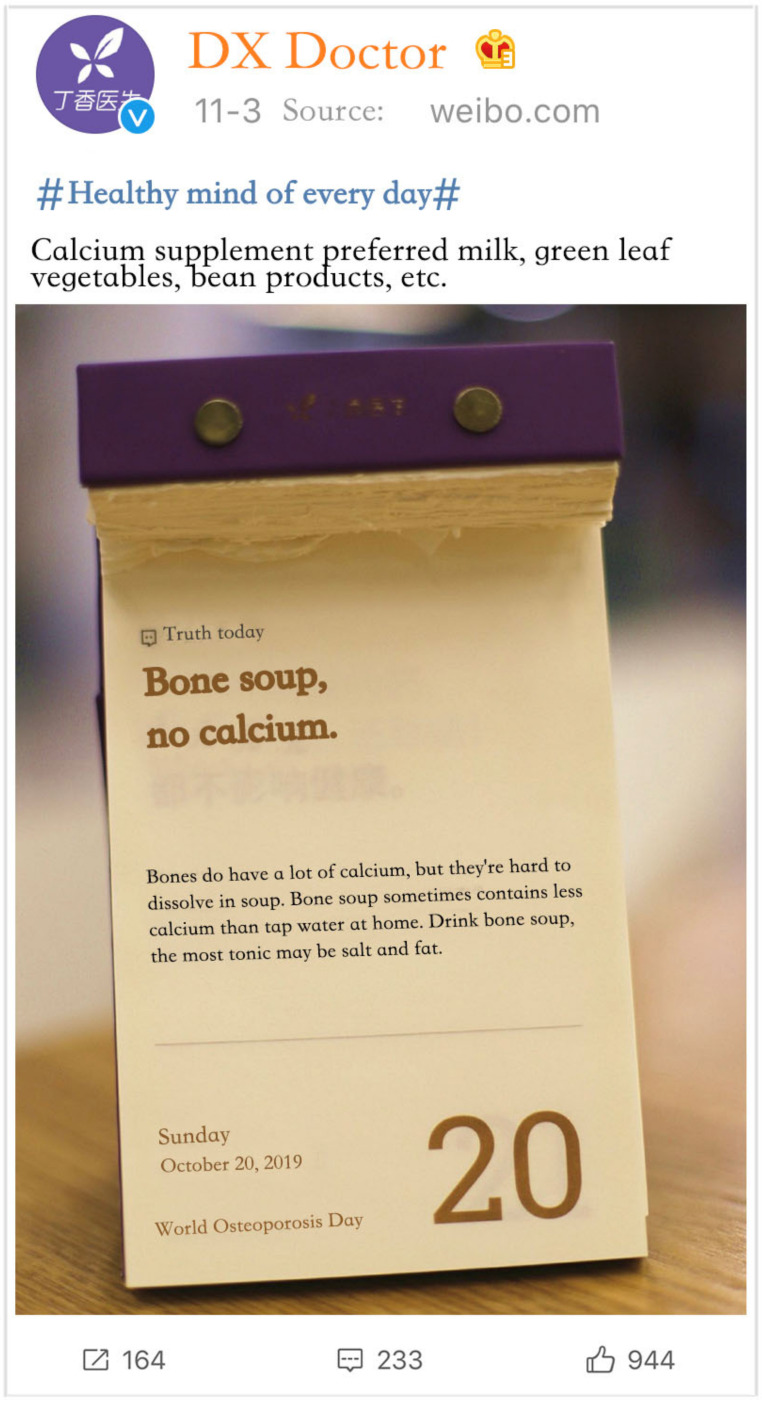
Health misinformation rebuttal.

In the second part, a situational questionnaire was used to describe specific response situations to the participants through pictures and texts. This allowed participants to imagine themselves in the situation and their responses to be subsequently measured. This method can reduce the influence of biases caused by factors, such as memory and comprehension. Figure 2 shows the health misinformation rebuttals to the respondents. We asked respondents to answer questions based on their actual perceptions of the experimental situation. For example, the respondents’ perceptions of source credibility were mainly their perceptions of the credibility of Sina Weibo and DX Doctor, and this differed between respondents.

The third part included the measurement items for each variable. The present scale was mainly derived from the mature measurement scale in the existing literature. The initial scale for the study situation in this study was designed based on the characteristics of health misinformation rebuttals and was improved through pre-investigation procedures (see Table 1). A score of one to seven was given ranging from total negative to total positive.

For data collection, we utilized So Jump, an online questionnaire survey platform that randomly sends the questionnaire to users, and eventually collected 415 valid questionnaires from 22 November to 3 December 2019. To ensure the quality of the questionnaire, we paid the respondents. There were 166 men (40%) and 249 women (60%). Regarding the age distribution, most respondents (i.e., 384) were aged between 18 and 40 years, accounting for 92.53% of the total sample size. Most of the respondents had bachelor’s degrees and belonged to various industries. The demographics for the research sample are presented in Table 2.

## 4. Data Analysis and Results

### 4.1. Non-Response Bias

Non-response bias refers to the fact that a respondent’s failure to answer the questionnaire due to various reasons may lead to bias in the research results. Armstrong and Overton (1977) argued that late responders are more likely to be similar to non-responders than early responders [55]. This study compared whether there were significant differences in occupation and education between early respondents (207 respondents who completed the questionnaire first) and later respondents (208 respondents who completed the questionnaire later). The independent sample t-test results showed no significant difference between the early and later stage respondents in occupation and education (*p* > 0.05) [56]. This indicates that the non-response bias in this study was not obvious and could be ignored.

### 4.2. Common Method Bias

When all data are from the same questionnaire, there may be common method bias, which affects the effectiveness of the study. This study examined the potential existence of common method bias through several procures. First, Harman’s single factor test was used. The results showed that the first (largest) factor accounted for 35.883% of the variance, no single factor explained more than 40% of the variance, and all factors explained 73.358% of the variance [57]. Then, the marker variable method was used to add a variable theoretically unrelated to other latent variables to the model [58,59]. The test showed that the label variable has no significant influence on the variables in the original model. Therefore, common method bias was not a key issue in this study.

### 4.3. Assessment of Reliability and Validity

We tested the indicator reliability, convergent validity, and discriminant validity to ensure the measurement results were reliable and valid. Reliability was verified through Cronbach’s alpha (CA) and composite reliability (CR). As shown in Table 3, CA and CR were both larger than 0.7, denoting high reliability of the data [60].

Convergent validity was evaluated through item loadings, CR, and average variance extracted (AVE). As illustrated in Table 3 and Table 4, the values of item loadings and CR were larger than 0.7, and the AVE values were larger than 0.5, meaning the data had satisfactory convergent validity [61].

Discriminant validity was appraised by comparing the square root of the AVE of each construct to the inter-construct correlations, and by comparing the item loadings to the cross-loadings. Moreover, the heterotrait-monotrait ratio (HTMT) is usually no more than 0.85, and when the concepts of perspectives are similar, the HTMT threshold can be extended to 0.90 [62]. Table 5 shows that the square root of the AVE of each construct was greater than the inter-construct correlations. Similarly, Table 4 depicts that all item loadings were higher on their factor than on any other factor. Table 6 shows that most HTMT was no more than 0.85, and all HTMT was no more than 0.90. These results confirmed the discriminant validity.

### 4.4. Assessment of the Structural Model

**Figure 3:**
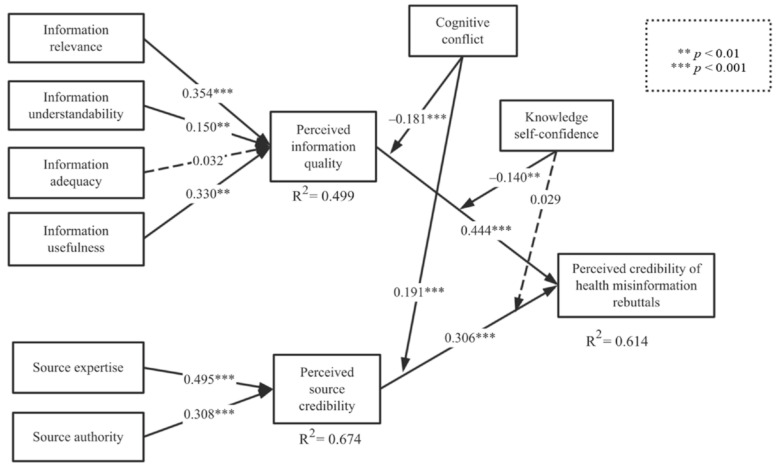
Results of the research model.

The results of the hypotheses based on the t-values, confidence intervals, and value of f-squared are presented in Table 7. The path coefficients and explained variance of the structural model are revealed in Figure 3. The $Q^{2}$ of the perceived credibility of rebuttals, perceived information quality, and perceived source credibility were 0.469, 0.334, and 0.442, respectively. The $R^{2}$ of the perceived credibility of rebuttals, perceived information quality, and perceived source credibility were 0.614, 0.499, and 0.566, respectively. The structural model showed high prediction accuracy. The perceived information quality (β=0.444,p<0.001) on the central route and perceived source credibility (β=0.306,p<0.001) on the peripheral route significantly affected the perceived credibility of the rebuttals of health misinformation. Hypotheses 1 and 2 were thus supported.

Further, information relevance (β=0.354,p<0.001), understandability (β=0.150,p<0.01), and usefulness (β=0.330,p<0.01) all significantly affected perceived information quality; however, the impact of information adequacy remained insignificant (β=0.032,p=0.617). Thus, Hypotheses 1a, 1b, and 1d were also supported, while hypothesis 1c was rejected. Additionally, information source expertise (β=0.495,p<0.001) and authority (β=0.308,p<0.001) had a significant impact on perceived source credibility; therefore, Hypotheses 2a and 2b were supported. Cognitive conflict negatively moderated the impact of perceived information quality on perceived information credibility (β=−0.089,p<0.05), and positively moderated the impact of perceived source credibility on perceived information credibility (β=0.135,p<0.01). Hypothesis 3a and 3b were consequently supported. Moreover, knowledge self-confidence played a negative moderating role in the relationship between perceived information quality and perceived information credibility (β=−0.140,p<0.01). However, its moderating role in the relationship between source credibility and perceived information credibility was insignificant (β=0.029,p=0.581), suggesting that Hypothesis 4a was valid, while Hypothesis 4b was invalid.

## 5. Discussion

The perceived credibility of health misinformation rebuttals can be improved from two aspects: information content itself and information source. In addition to authoritative experts having credibility recognized more by the public, they have a stronger ability to produce high-quality debunking information. Under some circumstances, the influence of the information itself on information credibility may be more important than the information source. Moreover, under circumstances wherein the harmful effects of health misinformation are relatively weak (not enough to attract the attention of authorities and many experts), the science involved in correcting health information is relatively simple, or it is difficult to verify that information through practice (e.g., measurement of calcium content in bone soup). Cho et al. (2011) found that information from different sources may have the same influence, for example, whether the recipient is told that the information comes from research funded by “ExxonMobil” or “people like you” [63]. These findings suggest that the factors of source reliability may be overlooked sometimes. Additionally, the crux of the information is often easier to remember than the source, and compelling stories from unreliable sources may be recalled or accepted long after the sources are forgotten [4]. However, it is difficult to accurately judge whether the health information is credible or not based only on the information content itself [64]. Focusing on the information source can reduce the sharing of false information [65]. Nonetheless, the combined effect of information quality and source reliability can enhance information credibility to a greater extent. Caulfield recommended that accurate information of value to the public must be shared and called on scientists to participate in science-related communication on social media [66].

Information quality can be improved by enhancing information relevance (i.e., meeting the audience’s demands and attracting the public’s interest), understandability (i.e., ensuring rational logic and high readability), and usefulness (i.e., helping the public solve problems with practically and feasibly) [29,32]. Public demand for information adequacy is not high. Information overload, similar to information scarcity, can be a hindrance; therefore, a full and comprehensive exposition of information is not necessary in some cases. People should take action to disseminate high-quality information globally that is accurate, easy to digest, engaging, and easy to share on mobile devices. Information must be customized to the recipient, since different people can perceive the same things in dramatically different ways. Encouraging individuals with large numbers of followers to share corrective or high-quality information and encouraging scientists to communicate more with the public on social media platforms may be effective strategies for combating false health information online.

The overwhelming information deluge on social media has affected the public’s understanding, and it is particularly significant to make use of expert resources to ensure that high-quality misinformation rebuttals stand out. The credibility of information sources often enhances the persuasiveness of communication. Ideally, the public should maintain a high level of trust for reliable sources, while trust in unreliable sources should be reduced. In practice, however, it often is difficult for the public to determine a source’s reliability. When people are confused about who can provide accurate information, it would be helpful to provide users with clearer indicators of the reliability of a source. Therefore, social media could rate information sources, such as through expert ratings (expert ratings of articles) and user rating of articles or information sources. Messages are popular mainly because influencers share them with their audiences [67]. Communicators with high credibility should pay attention to the authenticity of health information before they disseminate it to their followers, as individuals and businesses with a large social media audience have a greater responsibility to verify the accuracy of any health information they share. They should refer to points from real medical reports and authoritative experts, only forward information from trusted health knowledge providers, and make full use of the authority effect and platform effect to improve information credibility.

Although the cognitive conflict of the information receiver has a significant moderating role in the two paths of information quality and source credibility, the moderating effects are completely different. When cognitive conflict is high, the influence of information quality on perceived information credibility is hampered, while the effect of source credibility on perceived information credibility is enhanced. People evaluate the logical compatibility of the information they receive with other facts and beliefs. Once information is accepted, it is highly impervious to change. From the perspective of cognitive consistency, this resistance stems from subsequent inconsistencies in the information that result from the refusal to admit that previous information is false. Thus, conflict with existing knowledge reduces the likelihood that it will be successfully corrected. If there is insufficient evidence that the original perception is wrong, the easiest way to resolve this conflict may be to revert to the original idea and ignore the corrective input.

In general, people tend to hold on to what they already know. Changing perceptions entails additional motivation and cognitive resources, and if the topic is not of interest to the public, it is very difficult to change predominant misconceptions [4]. Therefore, for the receivers of information with high cognitive conflict, only high-quality persuasive arguments can convince them to change their original attitudes and beliefs, such as solid evidence and logic of eloquence. At this point, it is easier to persuade a person or medium that is highly trusted by the receiver of the message to disprove the misinformation. Evidently, inconsistent information can trigger negative emotions and increase the difficulty for the receiver to process the information. Nonetheless, trust in an information sender can supplement positive emotions without any effort to process the information, and it can be easily gauged by the marginal clue of source credibility.

The knowledge self-confidence of information receivers is not significant in moderating the relationship between source reliability and perceived information credibility, but it weakens the influence of information quality on perceived information credibility. Moreover, higher cognitive receivers need higher-quality persuasive arguments to persuade them to change their original beliefs. Contrariwise, this phenomenon may be attributable to the professionalism of health information. Hence, an audience with higher knowledge self-confidence trusts its original cognition or professional level but does not exclude that which it believes is inaccurate. Reliance on false knowledge is not the same as ignorance, which is lack of relevant knowledge. Ignorance can have significant adverse effects on decisions, but these effects may not be as severe as the impact of trust in false knowledge. When people lack knowledge, they often rely on simple heuristics for making decisions. Overall, they have a relatively low level of confidence in decisions based solely on heuristics [68,69]. In other words, ignorance rarely leads to strong support for an idea, but if there is a high level of confidence in one’s knowledge, this support is often powerful. For example, individuals who oppose the scientific evidence for climate change most strongly are usually those who think they are experts on the subject [70]. Pre-existing scientific knowledge may influence the interpretation of newly received scientific knowledge [4]. When individuals are confident in their knowledge, but they cannot make a reasonable explanation with their original knowledge, they may be more difficult to be persuaded. This may be because an audience with high knowledge self-confidence has higher requirements regarding scientific explanations and information quality.

The public should be aware of the limitations of its cognition, and confidence in existing knowledge should not be an obstacle to new ideas. Social media should pay close attention to the “water army” of the Internet, use strict measures to prevent misinformation from spreading, and increase the speed at which misinformation rebuttals are spread by changing the manner in which information flow is presented. Thus, with these changes, the public could receive correct information before being subjected to misinformation.

## 6. Conclusions

To address health misinformation and enhance public health, the results of this study suggest that perceived credibility of health misinformation rebuttals can be improved by enhancing the information quality and information source. Information quality has a stronger influence on information credibility than information source under some circumstances. Information quality can be improved by enhancing information relevance, understandability, and usefulness. Source reliability can be improved by enhancing source expertise and authority. However, the cognitive conflict and knowledge self-confidence of information receivers weaken the influence of information quality on information credibility. In contrast, cognitive conflict can strengthen the influence of source credibility on information credibility. The public should be aware of the limitations of its cognition. Governments, platform managers, medical and health service personnel, and educators should combine the effect of information quality and source reliability to enhance information credibility.

This study constructed a theoretical model of the factors influencing the perceived credibility of health misinformation rebuttals on social media. First, this study analyzed the influencing factors from three perspectives, including information, information source, and the information recipient. Not only will this study enrich the application of the ELM theory but also expand the governance and mitigation of misinformation on social media. Second, information quality and source credibility are cardinal to changing the public’s perception. As it is more difficult to correct existing misinformation than accept new knowledge, the model in this study is a theoretical trial carried out solely to refute health misinformation on social media. Lastly, the target audience of the rebuttal of health misinformation has high cognitive conflicts and widely different knowledge self-confidence. This study focused on analyzing the moderating roles of the knowledge self-confidence and the cognitive conflict on correction paths. It is a theoretical complement to the persuasion field and is of great significance for understanding the public’s attitudes and changing their behaviors.

In practice, this study is of significance for the government, platform managers, medical and health service personnel, and educators. First, information quality and source credibility play a prime role in changing the public’s belief. Information publishers should provide high-quality, understandable, useful, and convincing information. Besides, the information should be transmitted by highly reliable transmitters, including the government or authoritative medical institutions. This will help enhance the public’s perception of information credibility. Second, the existence of cognitive conflict increases the difficulty of refuting misinformation. Misinformation refutation by credible communicators, such as governments and authoritative medical experts trusted by information receivers, can get twice the result with half the effort. Social media platforms that share health information should control the quality of information and identify and filter unhealthy information in a timely manner. Moreover, credit rating and other markers are used to distinguish the credibility of information publishers and reduce the difficulty in users’ cognitive processing. Lastly, users have different degrees of understanding of health professional information, and users with high knowledge self-confidence are the groups that find it more difficult to change their attitude. On the one hand, social media platforms can obtain correct information before users are exposed to health misinformation by means such as changing information presentation order. On the other hand, they need to push high-quality misinformation rebuttals from different sources more frequently to the hard-to-persuade groups.

This research has some limitations that should be overcome to expand future studies. First, it studied representative factors for the information, information source, and receiver. Notably, the effect of correcting health misinformation is influenced by numerous factors. Thus, more effort should be made in discovering other factors to improve the model—that is, the model can be combined with psychological factors to explore the psychological process of the public’s acceptance or rejection of the beliefs change and with sociological factors to explore solutions to the echo chamber situation. Second, we studied a single situation (i.e., bone soup, no calcium) through a questionnaire survey. The obtained results were based on the specific set up of the survey, and the applicability of the suggestions to other scenarios needs further consideration. Hence, other situations can be designed to study health misinformation according to the standards for levels of risks or different social and cultural backgrounds. Further, behavioral experiments can be conducted to manipulate signals about source credibility, social identity, and perceived importance of the information itself.

## Figures and Tables

**Table 1:** Measurement items and sources.

Constructs	Measurement Items	Sources
Information Relevance	The information applies to my needs.	
	The information is relevant to me.	
	How much are you interested in the information?	
Information Understandability	The information is clear in meaning.	[32]
	I think the information is easy to read.	
	I think the information is understandable.	
Information Adequacy	I think the health message provides complete information.	
	I think the health message provides adequate information.	
	I think the health message provides sufficient information.	
Information Usefulness	I think the information is informative.	
	I think the information is helpful.	
	I think the information is useful.	
Perceived Information Quality	I think the information has high quality.	[15]
	I think the information is valuable.	
	I think the information is meaningful.	
Source Expertise	I think the information provider is an expert on this topic	[53]
	I think the information publisher is familiar with related knowledge.	
	I think the information publisher has the qualifications for publishing speeches about the topic.	
Source Authority	I think the information publisher is influential.	[21]
	I think the information publisher is reputed.	
	I think the information publisher is authoritative.	
Perceived Source Credibility	I think the source of the information is reliable.	[53]
	I think the source of the information is dependable.	
	I think the source of the information is trustworthy.	
Cognitive Conflict	This is the degree to which the information differs from what you already know.	[15,54]
	This is the degree to which this information conflicts with your prior knowledge.	
	This is the degree to which this information is inconsistent with your original perception.	
Knowledge Self-confidence	How much do you know about related knowledge?	[24]
	How much are you familiar with related knowledge?	
	How quickly do you grasp related knowledge?	
Perceived Credibility of Health Misinformation Rebuttals	I think the information is credible.	[21]
	I think the information is authentic.	
	I think the information is believable.	

**Table 2:** Demographic information of study participants.

Demographic Category	Number (*N* = 415)	Percentage (%)
Gender		
Men	166	40%
Women	249	60%
Age (years)		
<18	4	0.96%
18−25	103	24.82%
26–30	126	30.06%
31−40	155	37.35%
>40	23	6.51%
Education		
High school or below	8	1.93%
Bachelor’s degree	379	91.32%
Master’s degree	26	6.27%
Doctoral degree	2	0.48%

**Table 3:** Cronbach’s alpha (CA), composite reliability (CR), and average variance extracted (AVE).

Constructs	CA	rho A	CR	AVE
Information Relevance	0.747	0.763	0.856	0.665
Information Understandability	0.768	0.786	0.865	0.681
Information Adequacy	0.857	0.859	0.913	0.777
Information Usefulness	0.848	0.852	0.908	0.766
Perceived Information Quality	0.800	0.802	0.882	0.714
Source Expertise	0.799	0.804	0.882	0.713
Source Authority	0.775	0.814	0.866	0.683
Perceived Source Credibility	0.895	0.896	0.935	0.827
Cognitive Conflict	0.913	0.920	0.945	0.852
Knowledge Self-confidence	0.880	0.893	0.926	0.807
Perceived Credibility of Health Misinformation Rebuttals	0.885	0.887	0.929	0.813

**Table 4:** Item factor loadings and cross-loadings.

	IR	IUN	IA	IUS	PIQ	SE	SA	PSC	CC	KC	PIC
IR1	0.842	0.304	0.331	0.403	0.508	0.304	0.358	0.451	−0.120	0.026	0.442
IR2	0.863	0.307	0.460	0.489	0.533	0.367	0.375	0.513	−0.128	0.093	0.469
IR1	0.736	0.303	0.310	0.394	0.413	0.472	0.401	0.438	0.041	−0.011	0.253
IUN1	0.407	0.849	0.475	0.499	0.454	0.404	0.367	0.378	0.019	−0.100	0.250
IUN2	0.249	0.844	0.329	0.456	0.383	0.349	0.367	0.306	−0.045	−0.104	0.230
IUN3	0.240	0.781	0.274	0.383	0.326	0.266	0.224	0.202	−0.086	0.022	0.222
IA1	0.364	0.407	0.862	0.398	0.345	0.292	0.359	0.403	−0.073	0.070	0.295
IA2	0.426	0.395	0.884	0.467	0.377	0.439	0.392	0.510	−0.055	0.112	0.367
IA3	0.409	0.381	0.899	0.382	0.377	0.348	0.365	0.409	−0.072	0.085	0.335
IUS1	0.463	0.497	0.444	0.893	0.543	0.648	0.549	0.686	−0.159	0.070	0.480
IUS2	0.518	0.466	0.421	0.888	0.568	0.639	0.567	0.650	−0.093	0.048	0.459
IUS3	0.397	0.472	0.372	0.846	0.497	0.569	0.462	0.612	−0.117	0.007	0.420
PIQ1	0.538	0.376	0.409	0.528	0.834	0.531	0.514	0.598	−0.214	0.122	0.665
PIQ2	0.482	0.413	0.303	0.536	0.860	0.429	0.439	0.529	−0.210	0.053	0.587
PIQ3	0.491	0.421	0.336	0.487	0.840	0.384	0.437	0.475	−0.212	0.075	0.517
SE1	0.438	0.265	0.306	0.589	0.410	0.876	0.625	0.650	−0.043	0.078	0.521
SE2	0.342	0.456	0.276	0.651	0.468	0.823	0.628	0.559	−0.073	−0.027	0.418
SE3	0.374	0.356	0.451	0.561	0.484	0.833	0.647	0.624	−0.087	−0.026	0.408
SA1	0.295	0.298	0.272	0.438	0.414	0.572	0.815	0.467	−0.146	0.016	0.344
SA2	0.304	0.377	0.272	0.455	0.475	0.586	0.809	0.467	−0.075	0.023	0.432
SA3	0.492	0.315	0.455	0.574	0.475	0.682	0.855	0.694	−0.003	−0.058	0.486
PSC1	0.541	0.346	0.448	0.663	0.599	0.690	0.651	0.902	−0.174	0.047	0.569
PSC2	0.494	0.315	0.429	0.666	0.538	0.631	0.584	0.919	−0.190	0.082	0.555
PSC3	0.527	0.342	0.487	0.694	0.595	0.657	0.615	0.907	−0.220	0.096	0.606
CC1	−0.085	−0.026	−0.079	−0.117	−0.247	−0.064	−0.087	−0.183	0.911	−0.409	−0.334
CC2	−0.097	−0.026	−0.072	−0.144	−0.254	−0.080	−0.065	−0.228	0.938	−0.400	−0.345
CC3	−0.078	−0.057	−0.056	−0.126	−0.188	−0.076	−0.064	−0.177	0.921	−0.399	−0.291
KC1	0.003	−0.088	0.067	−0.001	0.086	−0.026	−0.013	−0.002	−0.418	0.918	0.220
KC2	0.078	−0.034	0.087	0.085	0.098	0.033	−0.034	0.085	−0.375	0.926	0.206
KC3	0.056	−0.100	0.129	0.052	0.086	0.033	0.005	0.157	−0.383	0.848	0.174
PIC1	0.455	0.268	0.313	0.536	0.664	0.498	0.478	0.607	−0.325	0.237	0.905
PIC2	0.453	0.244	0.339	0.426	0.596	0.434	0.433	0.532	−0.329	0.216	0.903
PIC3	0.406	0.257	0.373	0.435	0.639	0.510	0.488	0.575	−0.299	0.153	0.897

Note: IR, Information relevance; IUN, Information understandability; IA, Information adequacy; IUS, Information usefulness; PIQ, Perceived information quality; SE, Source expertise; SA, Source authority; PSC, Perceived source credibility; CC, Cognitive conflict; KC, Knowledge self-confidence; PIC, Perceived information credibility.

**Table 5:** Latent variable correlations.

	IR	IUN	IA	IUS	PIQ	SE	SA	PSC	CC	KC	PIC
IR	0.815										
IUN	0.372	0.825									
IA	0.454	0.447	0.882								
IUS	0.527	0.546	0.472	0.875							
PIQ	0.598	0.476	0.416	0.614	0.845						
SE	0.457	0.419	0.410	0.708	0.535	0.844					
SA	0.459	0.395	0.422	0.603	0.551	0.750	0.826				
PSC	0.573	0.368	0.501	0.742	0.636	0.726	0.679	0.909			
CC	−0.095	−0.038	−0.075	−0.140	−0.251	−0.079	−0.078	−0.214	0.923		
KC	0.049	−0.081	0.102	0.049	0.100	0.012	−0.016	0.082	−0.436	0.898	
PIC	0.486	0.285	0.378	0.518	0.703	0.534	0.518	0.635	−0.352	0.224	0.902

Note: IR, Information relevance; IUN, Information understandability; IA, Information adequacy; IUS, Information usefulness; PIQ, Perceived information quality; SE, Source expertise; SA, Source authority; PSC, Perceived source credibility; CC, Cognitive conflict; KC, Knowledge self-confidence; PIC, Perceived information credibility.

**Table 6:** Value of the heterotrait-monotrait ratio.

	IR	IUN	IA	IUS	PIQ	SE	SA	PSC	CC	KC	PIC
IR											
IUN	0.479										
IA	0.605	0.617									
IUS	0.658	0.670	0.613								
PIQ	0.767	0.599	0.534	0.742							
SE	0.670	0.526	0.651	0.858	0.737						
SA	0.482	0.507	0.491	0.661	0.633	0.861					
PSC	0.700	0.430	0.624	0.851	0.745	0.884	0.685				
CC	0.148	0.074	0.091	0.159	0.291	0.099	0.130	0.235			
KC	0.081	0.120	0.113	0.077	0.117	0.084	0.054	0.107	0.486		
PIC	0.585	0.343	0.441	0.595	0.828	0.656	0.535	0.711	0.390	0.252	

Note: IR, Information relevance; IUN, Information understandability; IA, Information adequacy; IUS, Information usefulness; PIQ, Perceived information quality; SE, Source expertise; SA, Source authority; PSC, Perceived source credibility; CC, Cognitive conflict; KC, Knowledge self-confidence; PIC, Perceived information credibility.

**Table 7:** Results of the hypotheses testing.

	t	Confidence Intervals		$f^{2}$
		2.5%	97.5%	
H1	10.485	0.363	0.523	0.291
H1a	6.559	0.242	0.463	0.169
H1b	2.820	0.060	0.261	0.029
H1c	0.243	−0.124	0.152	0.000
H1d	3.430	0.126	0.512	0.122
H2	6.632	0.212	0.399	0.136
H2a	14.420	0.569	0.752	0.560
H2b	2.206	0.023	0.228	0.018
H3a	3.897	−0.270	−0.089	0.032
H3b	5.556	0.122	0.254	0.046
H4a	2.811	−0.232	−0.047	0.020
H4b	0.573	−0.073	0.120	0.001
